# Computer-aided design and synthesis of 3-carbonyl-5-phenyl-1*H*-pyrazole as highly selective and potent BRAFV600E and CRAF inhibitor

**DOI:** 10.1080/14756366.2019.1599366

**Published:** 2019-07-16

**Authors:** Jinwoong Kim, Byeongha Choi, Daseul Im, Hoyong Jung, Hyungwoo Moon, Waqar Aman, Jung-Mi Hah

**Affiliations:** a Department of Pharmacy and Institute of Pharmaceutical Science and Technology, College of Pharmacy, Hanyang University, Ansan, Gyunggido, Republic of Korea;; b Kohat University of Science and Technology, Kohat, Khyber Pukhtunkhwa, Pakistan

**Keywords:** Melanoma, BRAFV600E, BRAFWT, CRAF, selectivity

## Abstract

BRAF belongs to the upstream portion of the MAPK pathway, which is involved in cell proliferation and survival. When mutations occur in BRAF, downstream MEK and ERK are phosphorylated irrespective of RAS, resulting in melanoma-like cancer. Over the years, small molecules targeting BRAFV600E have been discovered to be very effective melanoma drugs, but they are known to cause the BRAF paradox. Recently, it was shown that this paradox is caused by the heterodimer phenomenon of BRAF/CRAF. Here, we suggest one method by which paradoxical activation can be avoided by selectively inhibiting BRAFV600E and CRAF but not wild-type BRAF. From previous report of *N*-(3-(3-alkyl-1*H*-pyrazol-5-yl) phenyl) aryl amide as a selective inhibitor of BRAFV600E and CRAF, we present compounds that offer enhanced selectivity and efficacy with the aid of molecular modelling.

## 1. Introduction

Malignant melanoma is the deadliest of all skin cancers. Historically, melanoma was very rare, but its prevalence has increased rapidly over the last 50 years compared to other cancers. Sunburn and congenital naevi are included among the risk factors of melanoma and may also be influenced by family history.^1^ At present, it is most effective to surgically remove lesion sites in patients with early melanoma after diagnosis, and lymph node dissection around the lesion is often considered because radiation or melanoma cells can be transferred to the surrounding lymph nodes. Immunotherapy with drugs such as nivolumab or ipilimumab or targeted chemotherapy for BRAF gene mutations is used to treat the surrounding lymph nodes in patients with metastatic melanoma.^2^

The RAS/RAF/MEK/ERK signaling pathway regulates cell growth, proliferation, and survival. RAF families act as critical modulators in this signaling pathway and RAS-induced dimerisation of RAF phosphorylates MEK1, and MEK2 activates the downstream cascade, subsequently phosphorylating ERK1 and ERK2.^3^ This signaling pathway is the most frequently mutated in human cancers, with about 50% of melanoma patients. BRAF is found in 10–70% of patients with thyroid cancer, less than 10% of patients with colorectal cancer, and in 3–5% of patients with non-small cell lung cancer (NSCLC).^4^ V600E is the most abundant mutation in BRAF; therefore, BRAF V600E is the most popular target of melanoma treatment.^5^

**Figure 1.**
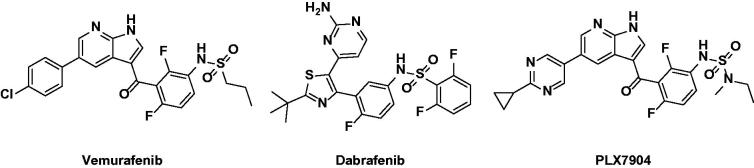
Chemical structures of BRAF paradox inducer (Vemurafenib, Dabrafenib) and breakers (PLX7904 and TAK-632).

The drugs vemurafenib (Zelboraf)^6^ and dabrafenib (Tafinlar)^7^ were developed and approved by the FDA as inhibitors targeting BRAFV600E for use in therapies for advanced melanoma treatment (Figure 1). These drugs are associated with significant response and enhanced survival rates in melanoma patients with BRAFV600 mutant tumors. However, resistance to these drugs often occurs within 1 year, and the disease then recurs. In addition, secondary malignant tumors arising from RAS mutations, such as squamous cell carcinoma and keratocyte sarcoma, may occur during treatment. BRAF inhibitors can bind to wild-type RAF and induce dimerization to phosphorylate downstream proteins regardless of RAS activation. This phenomenon is referred to as the BRAF paradox.^8^^,^^9^ The mechanism underlying this phenomenon is dimerization of RAF, which has emerged as a clinically important issue as vemurafenib and dabrafenib do not effectively inhibit homodimers or heterodimers of BRAF or CRAF. Another BRAF paradox mechanism is that first-generation BRAF inhibitors bind not only to wild-type BRAF, but also to one of the BRAF dimers, turning the αC-helix into an outward facing position. These inhibitors competitively bind to ATP sites in the DFG IN/αC-helix OUT conformation of BRAF as type I1/2 inhibitors. As a result, the αC-helix of another protomer is transformed into an OUT structure, preventing other inhibitors from binding. Downstream signaling is activated by unbound protomer. To prevent occurrence of the paradox, blocking mutations in RAS together with CRAF, one of the protein kinases of other RAF families, is effective, as is blocking BRAF mutations. Therefore, it is important to maintain CRAF as well as BRAFV600E.^10^

**Figure 2.**
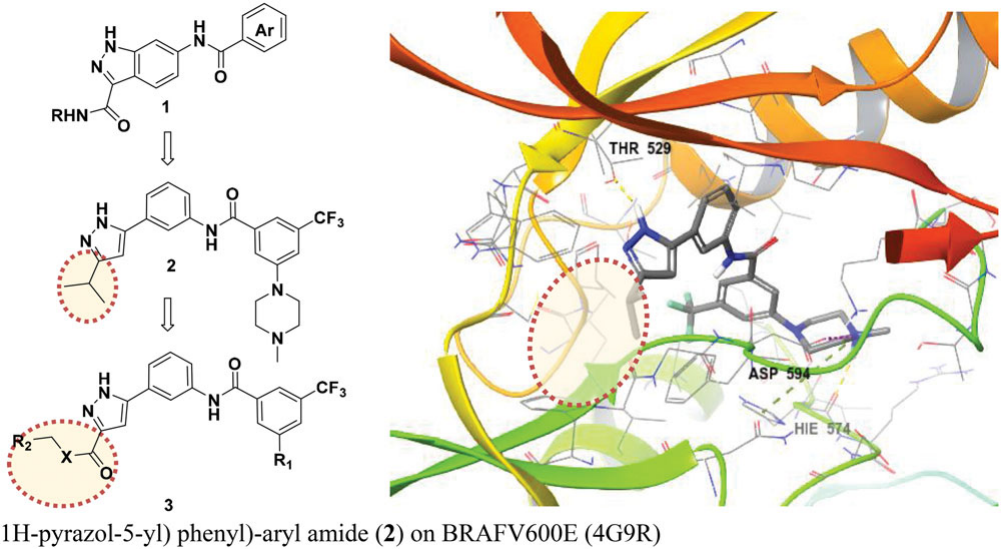
Selective BRAFV600E and CRAF inhibitors reported and docking mode of N-(3–(3-alkyl-1*H*-pyrazol-5-yl) phenyl)-aryl amide (2) on BRAFV600E (4G9R).

We have suggested a BRAF inhibitor that selectively inhibits BRAFV600E and CRAF but not BRAFWT for use as a BRAF paradox breaker and previously described 3-carboxamido-2*H*-indazole-6-arylamide (**1**), a selective CRAF inhibitor with an IC_50_ of 38.6 nM (Figure 2). However, the IC_50_ was only 7.82 μM for BRAFV600E and 9.45 μM for wild-type BRAF.^11^ As a follow-up study aimed at identifying selective binding inhibitors of BRAF V600E and CRAF, *N*-(3–(3-alkyl-1*H*-pyrazol-5-yl)phenyl)-aryl amide (**2**) were developed with good selectivity for BRAFV600E and CRAF.^12^

Here, we describe 3-carbonyl-5-phenyl-1*H*-pyrazole derivatives that strengthen activity. They were designed by extending the solvent exposure part in the ribose part of the scaffold through *in silico* modeling. 3-carbonyl-5-phenyl-1*H*-pyrazole has a binding mode that binds hydrogen with Cys 532 of the hinge and Thr529, which is a gatekeeper commonly found in BRAFV600E and CRAF. Glide docking of each compound was attempted to predict selectivity for BRAFV600E and CRAF over BRAFWT, thereby increasing potency while maintaining selectivity.

## 2. Results and discussion

Recently, we described *N*-(3–(3-alkyl-1*H*-pyrazol-5-yl)phenyl)-aryl amide (**2**) as a selective RAF inhibitor.^12^ This derivative showed very selective binding affinity to BRAFV600E and CRAF and was 20 and 126 times more potent than either, respectively, in BRAFWT. Structural features of these analogues include a bulky 1,3,5-substituted benzyl group in the type 2 hydrophobic pocket region and a common ring bond to pyrazole, the hinge binder, with a direct C-C bond. However, we found that the compound *N*-(3–(3-alkyl-1*H*-pyrazol-5-yl)phenyl)-aryl amide did not effectively hydrogen bond with the Cys531 of the hinge portion during glide docking in the Maestro programme. We conclude that this is the same problem seen in previous series, resulting in low potency compared to its high selectivity. A new inhibitor design was attempted by introducing an extra group capable of hydrogen bonding to hinge residues beside pyrazole. We assessed 3-carbonyl-5-phenyl-1*H*-pyrazole (**3**) as a new scaffold and performed docking studies.

**Figure 3.**
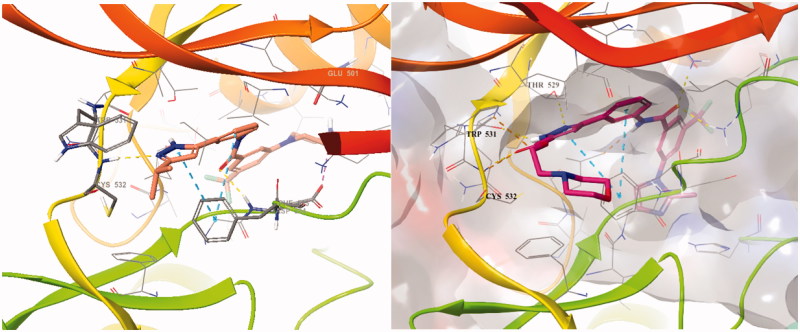
Binding mode of 2 and 3 to BRAFV600E (PDB: 4G9R).

To design better inhibitors in terms of activity and selectivity, we conducted docking studies to identify the best compound in a series of **2**, *N*-(3–(3-isopropyl-1*H*-pyrazol-5-yl)phenyl)-3-(4-methylpiperazin-1-yl)-5-(trifluoromethyl)benzamide. We then extended compound **2** to a 3-carbonyl-5-phenyl-1*H*-pyrazole (**3**) compound and tried to dock it to BRAFV600E (PDB Code: 4G9R)^13^ and CRAF (PDB Code: 3OMV)^14^ with Maestro (Figure 3). In general, the docking scores of series 3 were better than those of series **2** on a scale of 1–2 (10 to 100 times lower in energy). Compounds **2** and **3** were docked to BRAFV600E through induced fit docking and showed multiple interactions like those shown in Figure 3. Most importantly, 3-carbonyl-5-phenyl-1*H*-pyrazole (**3**) has three hydrogen bonds near the hinge site, with the proton of the pyrazole group hydrogen bonded to Trp531 and the amine of pyrazole to Thr529. The carbonyl group substituted at the pyrazole ring was also hydrogen bonded to Cys532 at the hinge. Moreover, Phe595 of the DFG motif and the pyrazole and middle phenyl rings of the compound have a π–π interaction. The carbonyl of amide is hydrogen bonded to Lys601 of the activation loop. Likewise, compound 3 maintained the interactions of compound **2**.

**Figure 4.**
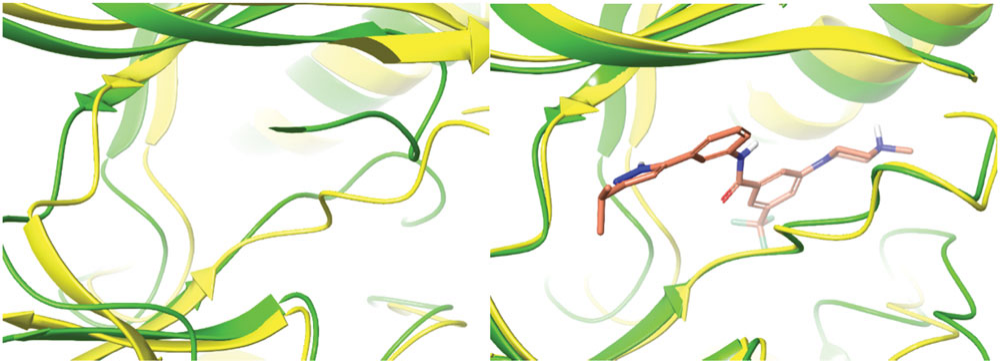
Overlapped binding mode between BRAFV600E (PDB: 4G9R, yellow) and CRAF (PDB: 3OMV, green), and overlapped binding mode between BRAFV600E (PDB: 4G9R, yellow) and modified CRAF (PDB: 3OMV, green).

The PDB for currently available CRAF is 3OMV, which is complexed with GDC0879 Reference. However, GDC0879 is a type 1 inhibitor for CRAF and binds the DFG motif of CRAF in the state of IN. Therefore, to explain the binding mode of our Type II inhibitors, we needed to modify 3OMV using the Induced Fit module in Maestro. First, we superimposed the binding mode of **2** to BRAFV600E (PDB: 4G9R) and to CRAF (PDB: 3OMV). Then, the positions of several residues around DFG in CRAF were shifted, rotated, and energy minimized as similarly as possible to the structure of BRAFV600E. We monitored the docking of compound **2** on CRAF each time we changed the structure. Through this process, a protein structure of CRAF for type 2 inhibitors was created, and the binding mode was confirmed, as shown in Figure 4. In induced fitted-CRAF and **2**, the pyrazole ring has a hydrogen bond with Cys424, and the hinge residue and amide bond were tied through two hydrogen bonds to the backbone of Asp486 of DFG. In addition, Phe487 and the pyrazole group of the DFG motif exhibit a π–π interaction, and the carboxylic acid of Glu393 of the N-lobe and proton of amide are hydrogen bonded. Finally, imidazole and N-lobe Lys375 undergo π–cation interactions, contributing to the stable binding of **2** to CRAF.

**Figure 5.**
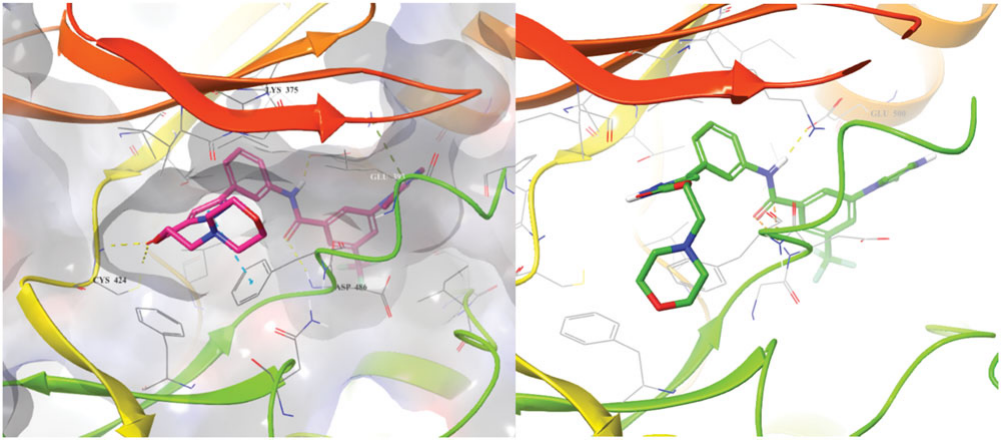
Binding mode of 23b to CRAF (PDB: 3OMV, glide docking) and to BRAFWT (PDB: 1UWH).^15^

We also attempted docking studies of compounds **2** and **3** on BRAFWT (Figure 5). In binding modes **2** and **3**, we observed only one hydrogen bond with Glu500 in the α-helix of BRAFWT, and hinge hydrogen bonds were not formed, resulting in a much lower docking score than for BRAFV600E (−9.813 to 1UWH, −15.128 to 4G9R). Most importantly, the difference in docking scores of BRAFV600E and BRAFWT was much greater for the new carbonyl series 3 than for series **2** (docking scores of series 3 on BRAFWT compared to BRAFV600E and CRAF were higher by magnitudes of 102–104).

After docking studies of 3-carbonyl-5-phenyl-1*H*-pyrazole (**3**) on three RAF proteins, we synthesized two kinds of carbonyl group-ester and amide-linked pyrazoles as new scaffolds under the assumption that inclusion of a hydrophobic tail would impart selectivity and potency that can be increased by extending the solvent exposure region.

**Scheme 1.**
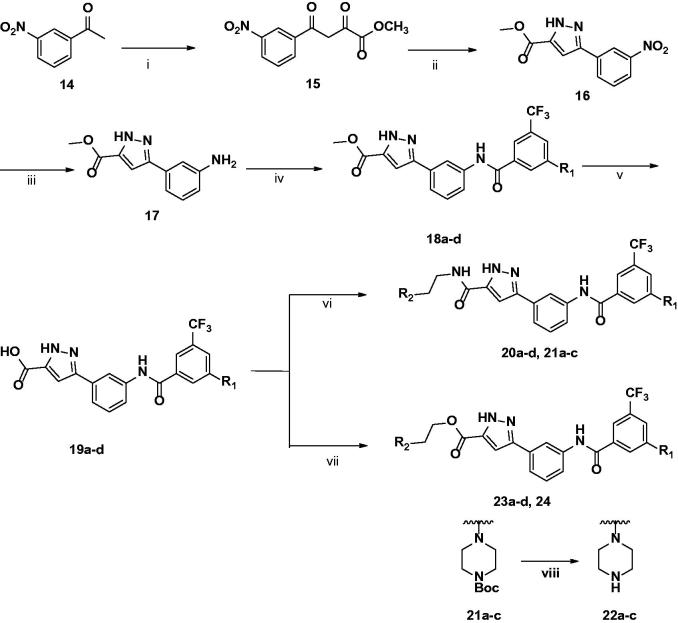
Synthesis of 3-carbonyl-5-phenyl-1*H*-pyrazole derivatives. (i) Diethyloxalate, NaOMe, MeOH, rt, 24h; (ii) NH_2_NH_2_·H_2_O, AcOH, rt, 4 h; (iii) H_2_, Pd/C, MeOH, rt, 30 min, 98%; (iv) benzoic acid, HATU, TEA, THF, rt, 24 h; (v) 1 M NaOH, THF, reflux, 2h; (vi) Alkylamine, HATU, DIPEA, THF, rt; (vii) Alkylalcohol, EDCI, HOBt, DMF, rt; (viii) TFA, DCM, rt, 30 min.

The general synthesis of 3-carbonyl-5-phenyl-1*H*-pyrazole (**3**) is shown in Scheme 1. The starting material 1-(3-nitrophenyl) ethan-1-one (**14**) was treated with a solution of sodium methoxide in methanol, and diethyl oxalate was added dropwise to yield **15**.^16^ Then, hydrazine monohydrate was reacted with **15** and acetic acid as a solvent at room temperature to obtain core intermediate pyrazole **16**.^17^ Sequentially, the nitro group of pyrazole **16** was reduced to aniline **17** through hydrogenation. Then, **17** was reacted with various benzoic acids with different substituents by amide coupling to obtain **18a**–**d**.^18^ After conversion of methyl ester (**18**) to its acid (**19**) using 1 M NaOH solution, the final amide products **20a**–**d** and **21a**–**c** were synthesised by amide coupling with several kinds of alkylamines, including HATU and DIPEA. The final ester products **23a**–**d** and **24** were synthesized by ester coupling with alkyl alcohols, EDCI, HOBt, or DMAP.^19^

The IC_50_ values for BRAFV600E and CRAF were measured for each of the 12 compounds synthesised (Table 1). The compounds were all designed as 1, 3, 5-substituted benzyl group hydrophobic tails to maintain the selectivity of the previous series.

**Table 1.**
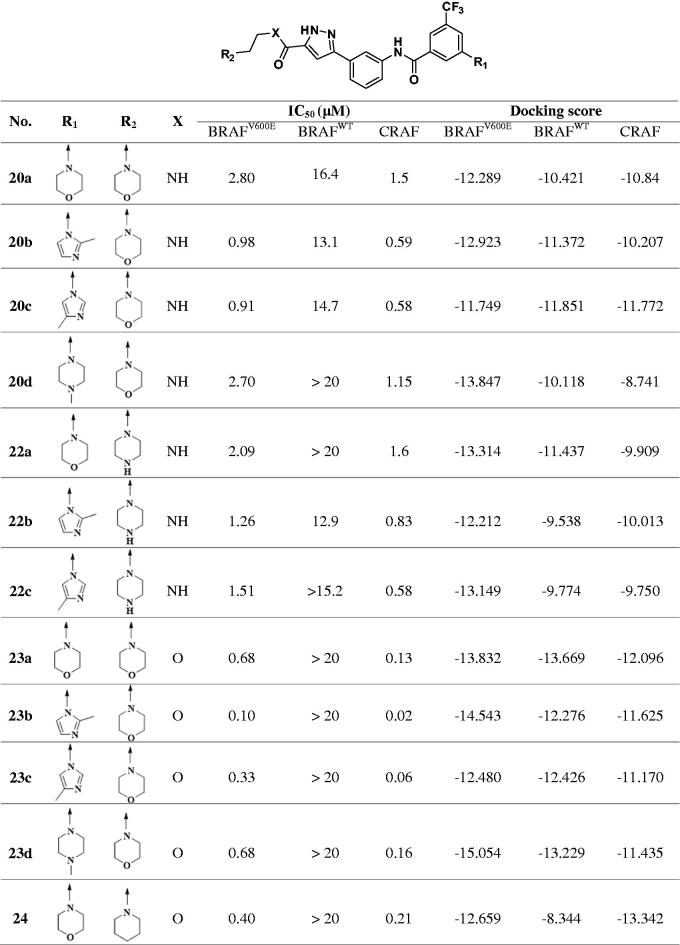
Enzymatic activities of 3-carbonyl-5-phenyl-1*H*-pyrazole derivatives.

For the R_1_ group of the hydrophobic tail, the IC_50_ values of the compounds increase in the order 2-methylimidazole < 4-methylimidazole < morpholine = 4-methylpiperazine. The ester linkage of the pyrazole group, which is an extended hinge binder and the part exposed to solvent, is superior to amide in terms of potency. Compounds that were substituted on the solvent exposure part with morpholine rather than piperazine showed lower IC_50_. Of these 12 compounds, **23b** showed the highest potency for BRAF^V600E^ and CRAF. Compound **23b** is a compound in which the hydrophobic tail is substituted with 2-methylimidazole, and the site exposed to the solvent is substituted with morpholine linked via ester. Compound 23 b is seven times more potent for BRAF^V600E^ and 5 times more potent for CRAF than the previously *N*-(3–(3-alkyl-1*H*-pyrazol-5-yl)phenyl)-aryl amide derivatives.

Next, we used kinase panel screening in duplicate for compound **23b** over 35 different kinases at a single-dose concentration of 10 μM (Table 2). The compound was indeed a selective BRAFV600E and CRAF inhibitor with an excellent selectivity profile. This compound has an inhibitory activity of more than 90% on CRAF and BRAF V600E at a concentration of 10 μM; the inhibition activity was less than 20% for most other kinases, including wild-type BRAF. We further determined the IC_50_ value of **23b** on wild-type BRAF to be over than 20 μM, where we could confirm selectivity (Table 3). In order to investigate the effect of **23b** in living cells, the human colon cancer cell line SW260 expressing wild-type BRAF and the malignant melanoma A375 cell line expressing BRAFV600E were treated with increasing concentrations of **23b.** The resulting MTT assays showed that in the cells expressing BRAFV600E (A375 cell line) **23b** resulted in inhibition of the proliferation (GI_50_ = 0.07 μM), and not in the cells expressing BRAFWT (SW620).

**Table 2.**
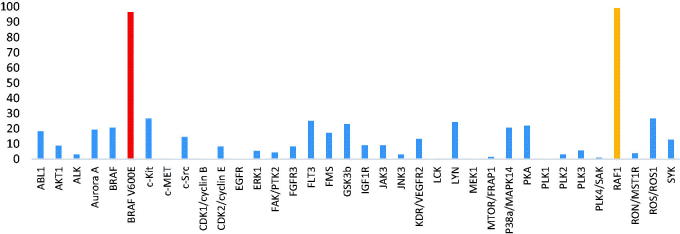
Percentages of enzymatic inhibition exerted by **23b** (10 μM) on 35 selected protein kinases

**Table 3.** Enzymatic activities of compound **23b**.

IC_50_	23b (μM)	Selectivity (fold)
BRAF^WT^	>20	–
BRAF^V600E^	0.10 (>200 fold)	>200
CRAF	0.02 (1000 fold)	>1000
A375^21^	0.07 μM (GI_50_)	
SW620^21^	>10 μM (GI_50_)	

## 3. Conclusions

We designed and synthesized selective, efficacious inhibitors for BRAFV600E and CRAF by *in silico* modeling using the Maestro programme.^20^ Various analogues obtained through computer-aided design were efficiently synthesised and measured for inhibitory activity against BRAFV600E and CRAF, respectively, and compound **23b** showed the best potency (BRAFV600E = 0.10 μM, CRAF = 0.02 μM) for each enzyme. This result is 7 times better for BRAFV600E and 5 times better for CRAF than for the previous series (BRAFV600E = 0.70 μM, CRAF = 0.11 μM). We attempted kinase panel screening for compound **23b** over 35 different kinases at a single-dose concentration of 10 μM (Table 2, performed in duplicate). The compound was, indeed, a selective BRAFV600E and CRAF inhibitor with an excellent selectivity profile. This compound has an inhibitory activity of 99% on BRAF V600E and CRAF at this concentration. The inhibition activity was less than 20% in most other kinases, including BRAFWT.

Considering that BRAFV600E and CRAF are significantly associated with disease progression and cell proliferation in a subset of melanomas and are potential paradox breakers, the finding of a dual RAF-kinase inhibitor, **23b**, was valuable. Our findings are significant for medicinal chemistry in two different ways. First, through *in silico* modeling, we were able to design highly selective and potent inhibitors for BRAFV600E and CRAF that were differentiated from similar BRAFWT. In addition, we confirmed the enzyme inhibitory activity and selectivity of the designed compounds. Second, we discovered a small molecule capable of more selective binding to BRAFV600E and CRAF than BRAFWT, suggesting a new direction for development of RAF paradox chemical inhibitors. Our findings also provide a way to improve the narrow therapeutic window of previous melanoma drugs, a particular disadvantage of pan-RAF inhibitors.
